# Both cell proliferation and apoptosis significantly predict shortened disease-free survival in hepatocellular carcinoma

**DOI:** 10.1038/sj.bjc.6690758

**Published:** 1999-10

**Authors:** Y Ito, N Matsuura, M Sakon, T Takeda, K Umeshita, H Nagano, S Nakamori, K Dono, M Tsujimoto, M Nakahara, K Nakao, M Monden

**Affiliations:** Department of Surgery II, Osaka University Medical School, Osaka, Japan; Department of Pathology, School of Allied Health Science, Faculty of Medicine, Osaka University, Osaka, Japan; Departments of Pathology, Surgery, Osaka Police Hospital, Osaka, Japan

**Keywords:** Ki-67, apoptosis, immunohistochemistry, hepatocellular carcinoma, prognostic factor

## Abstract

In this study, we investigated the proliferating cell index by the percentage of Ki-67 expressing cells (Ki-67 LI) and the apoptotic index (AI) by the number of morphologically apoptotic cells per 1000 carcinoma cells in haematoxylin and eosin sections of 76 hepatocellular carcinomas (HCC). Both indices showed excellent correlation with each other (*P* < 0.0001) and were significantly higher in cases of poor differentiation, of advanced stages, with portal invasion and with intrahepatic metastasis. Furthermore, cases with higher Ki-67 LI or higher AI displayed poor outcomes for disease-free survival (*P* = 0.0001 and *P* = 0.0005) by univariate analysis. By multivariate analysis, both indices could be regarded as independent prognostic factors. These results strongly suggest that Ki-67 LI and AI have very similar clinical significance, reflecting the existence of biologically aggressive phenotypes and poor disease-free survival rate in HCC. © 1999 Cancer Research Campaign


*British Journal of Cancer* (1999) **81**(4), 747–751
© 1999 Cancer Research Campaign
Article no. bjoc.1999.0758

### Both cell proliferation and apoptosis significantly predict shortened disease-free survival in hepatocellular carcinoma

**Y Ito[1,2], N Matsuura[2,1], M Sakon[1], T Takeda[1], K Umeshita[1], H Nagano[1], S Nakamori[1], K Dono[1], M Tsujimoto[3], M Nakahara[4], K Nakao[4] and M Monden[1]**

[1]Department of Surgery II, Osaka University Medical School, Osaka, Japan; [2]Department of Pathology, School of Allied Health Science, Faculty of Medicine, Osaka University, Osaka, Japan; Departments of [3]Pathology and [4]Surgery, Osaka Police Hospital, Osaka, Japan

**Summary** In this study, we investigated the proliferating cell index by the percentage of Ki-67 expressing cells (Ki-67 LI) and the apoptotic index (AI) by the number of morphologically apoptotic cells per 1000 carcinoma cells in haematoxylin and eosin sections of 76 hepatocellular carcinomas (HCC). Both indices showed excellent correlation with each other ($P < 0.0001$) and were significantly higher in cases of poor differentiation, of advanced stages, with portal invasion and with intrahepatic metastasis. Furthermore, cases with higher Ki-67 LI or higher AI displayed poor outcomes for disease-free survival ($P = 0.0001$ and $P = 0.0005$) by univariate analysis. By multivariate analysis, both indices could be regarded as independent prognostic factors. These results strongly suggest that Ki-67 LI and AI have very similar clinical significance, reflecting the existence of biologically aggressive phenotypes and poor disease-free survival rate in HCC. © 1999 Cancer Research Campaign

**Keywords:** Ki-67; apoptosis; immunohistochemistry; hepatocellular carcinoma; prognostic factor

Carcinoma progression and prognosis can be evaluated by many parameters. The simplest and most often used is the cell proliferating activity of the main tumours. In particular, Ki-67 is expressed by all cells except when they are at the G0 phase (Sasaki et al, 1987). Therefore, Ki-67 expression in carcinomas has been intensively investigated, and the Ki-67-positive cell rate has been shown to be significantly related to clinicopathological features and even survival time in cases of carcinoma of several organs (Avali-Lundqvist et al, 1997; Jansson et al, 1997; Molino et al, 1997; Victorzon et al, 1997; Beer et al, 1998; Komaki et al, 1998; Masuda et al, 1998). Another important phenomenon for evaluating the activity of carcinoma progression is cell death. In particular, apoptosis or programmed cell death, in carcinoma has been studied actively from various aspects since it became clear that carcinoma cells as well as normal cells can die with apoptosis. Previous studies have shown a high percentage of apoptotic cells correlated with high proliferating activity and/or shortened survival in carcinoma of bladder, uterus, ovary, lung, endometrium and prostate (Lipponen et al, 1994; Vasalainen et al, 1994; Heatley 1995; Tormanen et al, 1995; Todd et al, 1996; Yamasaki et al, 1997; Komaki et al, 1998), although there was one contrary result reported for colorectal carcinoma (Sugamura et al, 1998).

In the present study, we investigated both the Ki-67-positive cell rate and the morphologically apoptotic cell rate in hepatocellular carcinoma (HCC). As distant or lymph node metastases are usually not observed with HCC, we thought it very important to study the cell proliferation and cell death of the main tumour in order to evaluate the biological aggressiveness of this carcinoma.

*Received 20 October 1998*
*Revised 9 February 1999*
*Accepted 16 February 1999*

*Correspondence to:* N Matsuura, Department of Pathology, Allied Health Science, Osaka University Faculty of Medicine, 1–7, Yamadaoka, Suita, Osaka 565, Japan

### MATERIALS AND METHODS

### Tissue specimens

Ten per cent buffered formalin-fixed, paraffin-embedded blocks of HCC were prepared from 76 patients (65 males and 11 females: age 61.5 ± 9.9 years) who had undergone curative surgery for HCC diagnosed by computerized tomography, angiography and preoperative and intraoperative ultrasonography. Informed consent was obtained from each patient.

### Immunohistochemistry for Ki-67 and evaluation of Ki-67-labelling index

Immunohistochemical study was performed using the avidin–biotin complex (ABC) method. Briefly, 4-μm slices of tissue section were deparaffinized and endogenous peroxidase activity was blocked with 0.3% hydrogen peroxide and 0.1% sodium azide in distilled water for 15 min. The sections were then incubated with 0.03 mol $l^{-1}$ citrate buffer (pH 6.0) and heated to 121°C for 20 min in a pressure cooker. After three rinsings in phosphate-buffered saline (PBS) pH 7.2, 10% bovine serum (Wako, Osaka, Japan) was applied for 10 min to block the non-specific reaction. The sections were incubated with anti-Ki-67 monoclonal antibody (clone MIB-1, Ylem, Rome, Italy) at the concentration of 1:50 for 60 min at room temperature. After rinsing in PBS, they were treated with biotinylated sheep anti-mouse IgG (Amersham, London, UK) at the concentration of 1:50 for 15 min. Again, after rinsing in PBS, the sections were reacted with the avidin–biotin peroxidase complex (Dako, Denmark) at the concentration of

**Table 1** Relationship between clinicopathological parameters and Ki-67 LI and AI in 76 HCC cases

| | Ki-67 LI | AI |
|---|---|---|
| Tumour differentiation | | |
| Well or moderate (*n* = 55) | 21.8 ± 16.8 | 13.4 ± 6.6 |
| Poor (*n* = 21) | 47.7 ± 14.4 | 25.9 ± 10.9 |
| | *P* < 0.0001 | *P* < 0.0001 |
| Stage | | |
| I or II (*n* = 54) | 26.0 ± 19.4 | 14.7 ± 7.3 |
| III (*n* = 22) | 36.3 ± 19.3 | 22.0 ± 12.9 |
| | *P* = 0.0384 | *P* = 0.0023 |
| Portal invasion | | |
| With (*n* = 24) | 41.5 ± 20.2 | 21.7 ± 12.6 |
| Without (*n* = 52) | 23.2 ± 16.9 | 14.6 ± 7.3 |
| | *P* = 0.0001 | *P* = 0.0025 |
| Intrahepatic metastasis | | |
| With (*n* = 14) | 39.4 ± 19.3 | 22.3 ± 11.5 |
| Without (*n* = 62) | 26.6 ± 19.3 | 15.6 ± 9.0 |
| | *P* = 0.0274 | *P* = 0.0192 |

**Table 2** Univariate and multivariate analysis for disease-free survival

| | Univariate | Multivariate | | | |
|---|---|---|---|---|---|
| Tumour differentiation (well, moderate vs poor) | 0.0141 | 0.8757 | 0.8536 | – | – |
| Tumour size (5 cm or larger vs smaller than 5 cm) | 0.0148 | 0.4509 | 0.2727 | – | – |
| Portal invasion (+ vs –) | 0.0483 | 0.1929 | 0.0803 | – | – |
| Intrahepatic metastasis (+ vs –) | 0.0003 | 0.0346 | 0.1011 | – | – |
| Stage (I, II vs III) | 0.0005 | – | – | 0.0072 | 0.0056 |
| Ki-67 LI (20 or larger vs smaller than 20) | 0.0001 | 0.0088 | – | 0.0049 | – |
| AI (15 or larger vs smaller than 15) | 0.0005 | – | 0.0208 | – | 0.0016 |

1:300 for 15 min. The peroxidase reaction was visualized by incubating the sections with 0.02% 3,3′-diaminobenzidine tetrahydrochloride in 0.05 M Tris buffer (pH 7.6) with 0.01% hydrogen peroxide for 3 min. The sections were counterstained with haematoxylin. Sections for negative control were prepared by using normal mouse serum instead of primary antibody.

We counted Ki-67-positive cells by monitoring at least 500 HCC cells from at least five randomly selected fields. The percentage of Ki-67-positive cells was defined as the Ki-67-labelling index (LI).

### Evaluation of apoptotic index

We observed each section stained with haematoxylin and eosin (H&E), and regarded the cells as morphologically apoptotic when they satisfied two of the conditions by Kerr et al: eosinophilic cytoplasm, shrinkage of cell and pyknosis, and/or fragmentation of the nucleus (Kerr et al, 1972; Wyllie et al, 1980). We counted morphologically apoptotic cells by monitoring at least 1000 HCC cells from at least ten randomly selected fields. The number of apoptotic cells per 1000 cells was defined as the apoptotic index (AI).

### Survival data

Disease-free survival (DFS) of the 76 patients was followed from 6 to 83 months (mean 22.5 ± 17.1 months). Postoperative DFS curves were constructed by the Kaplan–Meier method.

### Statistical analyses

Values were expressed as mean ± s.e.m. The $\chi^2$ test and Student's *t*-test were adopted for analyses about the indices and clinicopathological parameters. The relationship between Ki-67 LI and AI was analysed by Spearman's rank correlation coefficient. Univariate DFS data were analysed by the log-rank test. For multivariate analyses, we used the Cox proportional hazard model. All *P*-values less than 0.05 were considered to be statistically significant.

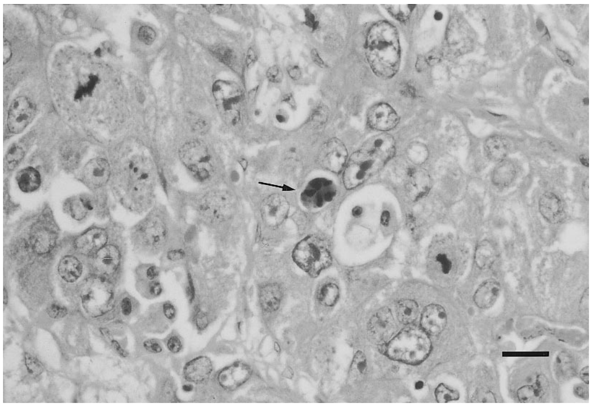

**Figure 1** Typical profile of morphologically apoptotic HCC cell in a haematoxylin–eosin-stained section (arrow). Scale bar: 20 μm

### RESULTS

We investigated Ki-67 LI and AI of HCC and their relationship with various backgrounds and clinicopathological features such as age, gender, viral infection, liver cirrhosis, tumour size, stage, degrees of differentiation, capsule formation, extracapsular invasion, septal formation, portal invasion and intrahepatic metastasis. Various pathological classifications including degrees of differentiation and stage were based on the classification of the Liver Cancer Group of Japan (1992).

Ki-67 LI of HCC in our series ranged from 3.0 to 81.5 (mean ± s.e.m., 29.1 ± 19.9). The indices were significantly higher in cases of poor differentiation ($P < 0.0001$), stage III ($P = 0.0384$), with portal invasion ($P = 0.0001$) and with intrahepatic metastasis ($P = 0.0274$) (Table 1). The number of cases with different degrees of differentiation, stage, portal invasion and intrahepatic metastasis are also noted in Table 1. The presence of portal invasion and intrahepatic metastasis were histologically determined.

Figure 1 shows a typical profile of morphologically apoptotic HCC cells in the H&E section. AI ranged from 4.5 to 44.5 (mean ± s.e.m., 17.2 ± 10.2) and a positive correlation was strongly observed in our cases between Ki-67 LI and AI ($P < 0.0001$) by Spearman's rank correlation coefficient. AI also

 *© 1999 Cancer Research Campaign*

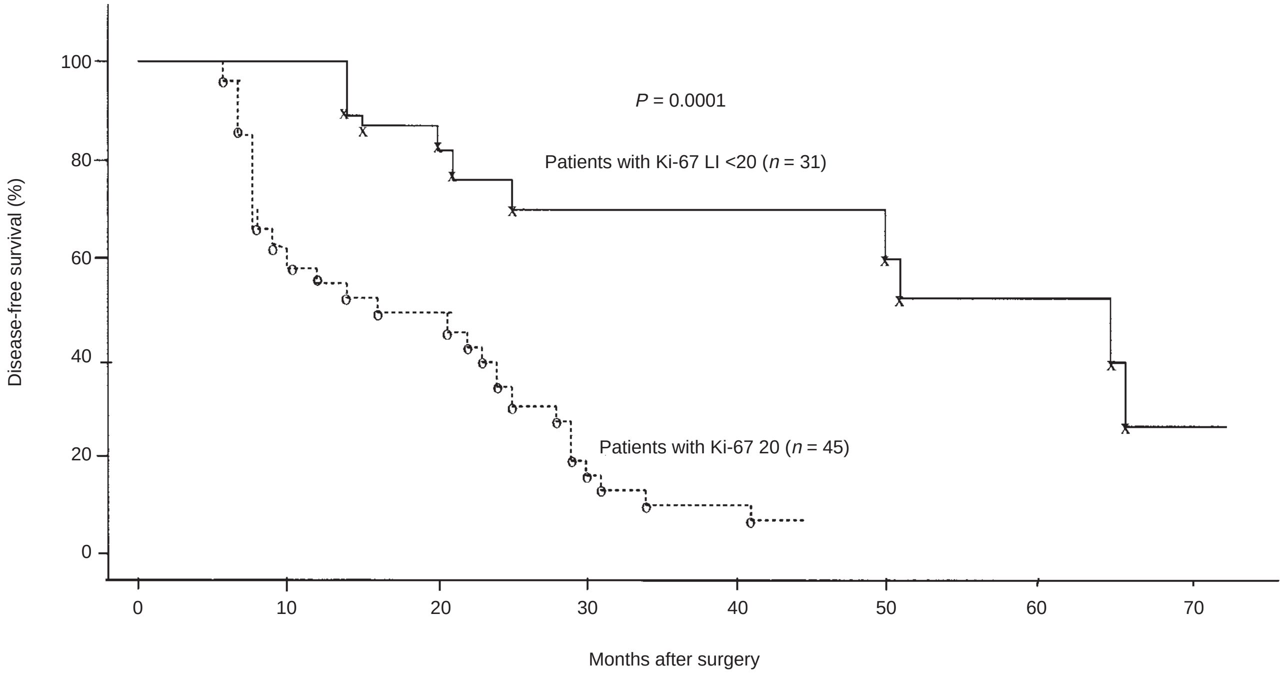

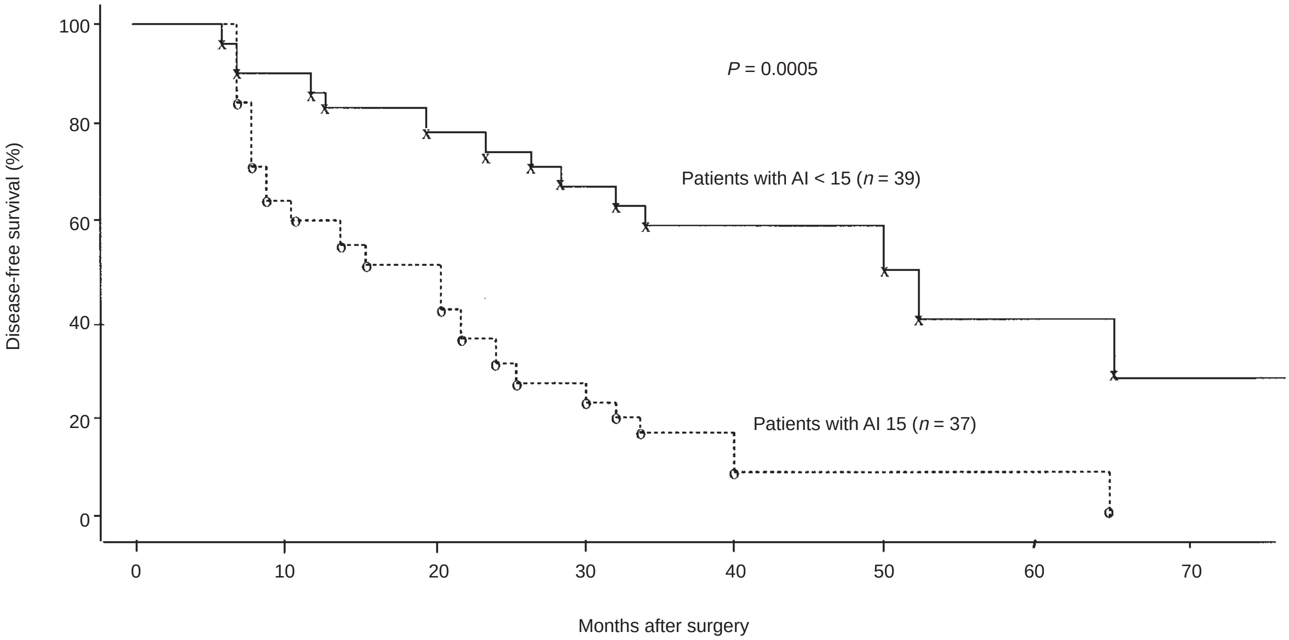

**Figure 2** (**A**) Disease-free survival curve of patients with Ki-67 LI ≥ 20 and < 20 following curative surgery. (**B**) Disease-free survival curve of patients with AI ≥ 15 and < 15 following curative surgery was significantly higher in cases of poor differentiation ($P < 0.0001$), stage III ($P = 0.0023$), with portal invasion ($P = 0.0025$) and with intrahepatic metastasis ($P = 0.0192$) as summarized in Table 1.

We then investigated the prognostic significance for DFS of each factor. The patients with Ki-67 LI higher than 20 and those with AI higher than 15 showed poorer outcomes for DFS ($P = 0.0001$ and $P = 0.0005$ respectively) by univariate analysis (Figure 2 A, B). We also performed multivariate analysis using the Cox proportional hazard model for each factor (Table 2) and found that, in addition to the clinical stage classification, Ki-67 LI and AI both served as independent prognostic factors for DFS of HCC patients after curative surgery. We performed the analysis by adding both Ki-67 and AI, but no informative results could be obtained probably because there is too much correlation between these two parameters.

### DISCUSSION

This study made it clear that Ki-67 LI is significantly higher in HCC cases with shorter DFS, as well as with biologically aggressive features such as poor differentiation, portal invasion, intrahepatic metastasis and advanced stages. Therefore, Ki-67 LI

*© 1999 Cancer Research Campaign*

should be a useful marker for evaluating the progressive activity and predicting DFS in HCC cases. Other groups have studied this. Three European groups could not establish the prognostic value for Ki-67 LI, probably because of the small number of cases in their studies (Colleoni et al, 1995; Faccioli et al, 1996; Nolte et al, 1998). Ng et al (1995) demonstrated that Ki-67 LI was correlated with carcinoma differentiation and DFS solely by univariate analysis. Although the antibody clone that they used was the same as ours, they heated the sections in citrate buffer in a microwave oven for antigen retrieval. This may have led to the results which differ from ours. We used a pressure cooker instead, because it can heat the sections more uniformly under higher temperature. In fact, for representative sections, we tried both methods and obtained higher Ki-67 LI when the pressure cooker was used (data not shown). The most recent study demonstrated the prognostic value of Ki-67 LI in HCC by multivariate analysis (King et al, 1998). However, it is strange that they could not establish any correlation between the LI and clinicopathological features, because cell-proliferating activity should contribute greatly to the biological aggressiveness of carcinoma. One possible reason for differing results is that they adopted a different clone from MIB-1 and used frozen sections.

We found a striking correlation between Ki-67 LI and AI ($P < 0.0001$), as well as positive relationships of AI with rapidly growing characters and even shorter DFS. These findings imply that the clinical significance of Ki-67 and AI in HCC are very similar, although they reflect two opposite phenomena: cell proliferation and cell death. Some groups have studied both apoptosis and cell proliferation in HCC (Hino et al, 1996; Soini et al, 1996; Zhao et al, 1997). Although these factors tended to show correlation in two studies (Hino et al, 1996; Zhao et al, 1997) using different markers, none of the reports could clarify the prognostic value of AI, probably because of the small number of cases examined.

Apoptosis in carcinoma is known to be induced by some kind of attack against carcinoma, such as by T lymphocytes by means of Fas ligand (Fas L) which binds to Fas located on the surface of carcinoma cells and turns them apoptotic (Itoh et al, 1991). However, Fas expression in HCC is reduced in biologically aggressive cases, such as poorly differentiated carcinoma (Ito et al, 1998), indicating a lesser likelihood of death by apoptosis by the Fas–Fas L pathway. Therefore, we hypothesize that there is another type of mechanism inducing apoptosis as a concomitant phenomenon to rapid growth and turnover of carcinoma cells. Fukuda et al (1993) demonstrated that apoptosis might be induced by, for example, autoregulation against the expanding population of carcinoma cells and/or cellular injury caused by hypoxia and nutrient deficiency (Fukuda et al, 1993). Although further studies are necessary, their findings may help to interpret, to some extent, our results about AI in HCC.

For detection of apoptotic cells, in-situ labelling of fragmented DNA is also widely employed (Gavrieli et al, 1992; Anasari et al, 1993; Gorczyca et al, 1993; Wijsman et al, 1993). However, previous studies reported that apoptotic cells could not be completely identified even by this method (Anasari et al, 1993; Gorczyca et al, 1993; Wijsman et al, 1993). Accordingly, morphological assessment of apoptosis using H&E-stained sections should still be very useful, especially in clinical fields, as the simplest method for detecting apoptotic cells (Gaffney et al, 1994; Heatley et al, 1995).

In summary, both Ki-67 LI and AI strongly reflect the biological characteristics of HCC and can be used to predict the DFS of HCC patients. As AI can be easily determined from H&E-stained sections, it should be extremely useful for screening the large number of HCC cases from various hospitals with various conditions of fixation.

### REFERENCES

Anasari B, Coates PJ, Greenstein BD and Hall PA (1993) In-situ end-labelling detects DNA strand breaks in apoptosis and other physiological and pathological states. *J Pathol* **170**: 1–8

Avali-Lundqvist EH, Silfversward C, Aspenblad U, Nilsson BR and Auer GU (1997) The impact of tumour angiogenesis, p53 overexpression and proliferative activity (MIB-1) on survival in squamous cervical carcinoma. *Eur J Cancer* **33**: 1799–1804

Beer TM, Buchanan R, Matthews AW, Stradling R, Pullinger N and Pethybridge RJ (1998) Prognosis in malignant mesothelioma related to MIB 1 proliferation index and histological subtypes. *Hum Pathol* **29**: 246–251

Colleoni M, Biasin MR and Boni L (1995) Evaluation of Ki-67 expression as a prognostic feature in hepatocellular carcinoma in cirrhosis. *Eur J Cancer* **31A**: 1547–1548

Faccioli S, Chieco P, Gramantieri L, Stecca BA and Bolondi L (1996) Cytometric measurement of cell proliferation in echo-guided biopsies from focal lesions of the liver. *Mod Pathol* **9**: 120–125

Fukuda K, Kojiro M and Jen-Fu Chu (1993) Demonstration of extensive chromatin cleavage in transplanted Morris hepatoma 7777 tissue: apoptosis or necrosis? *Am J Pathol* **142**: 2203–2207

Gaffney EP (1994) The extent of apoptosis in different types of high grade prostatic carcinoma. *Histopathology* **25**: 269–273

Gavrieli Y, Sherman Y, Shmuel A and Sasson B (1992) Identification of programmed cell death in situ via specific labeling of nuclear DNA fragmentation. *J Cell Biol* **119**: 493–501

Gorczyca W, Gong J and Darzynkiewicz A (1993) Detection of DNA strand breaks in individual apoptotic cells by the in situ terminal deoxynucleotidyl transferase and nick translation assays. *Cancer Res* **53**: 1945–1951

Heatley MK (1995) Association between the apoptotic index and established prognostic parameters in endometrial adenocarcinoma. *Histopathology* **27**: 469–472

Hino N, Higashi T, Nouso K, Nakatsukasa H and Tsuji T (1996) Apoptosis and proliferation of human hepatocellular carcinoma. *Liver* **16**: 123–129

Ito Y, Takeda T, Umeshita K, Sakon M, Wakasa K, Matsuura N and Monden M (1998) Fas antigen expression in hepatocellular carcinoma tissues. *Oncol Rep* **5**: 41–44

Itoh N, Yonehara S, Ishii A, Yonehara M, Mizushima S, Sameshima M, Hase A et al (1991) The polypeptide encoded by the cDNA for human cell surface antigen Fas can mediate apoptosis. *Cell* **66**: 233–243

Jansson A and Sun XF (1997) Ki-67 expression in relation to clinicopathological variables and prognosis in colorectal carcinomas. *APMIS* **105**: 730–734

Kerr JFR, Wyllie AH and Currie AR (1972) Apoptosis: a basic biological phenomenon with wide-ranging implications in tissue kinetics. *Br J Cancer* **26**: 239–257

King KL, Hwang JJ, Chau GY, Tsay SH, Chi CW, Lee TG, Wu LH, Wu CW and Lui WY (1998) Ki-67 expression as a prognostic marker in patients with hepatocellular carcinoma. *J Gastroenter Hepatology* **13**: 273–279

Komaki R, Miles L, Ro JY, Fujii T, Perkins P, Allen P, Sikes CR, Mountain CF and Ordonez NG (1998) Prognostic biomarker study in pathologically staged N1 non-small-cell lung cancer. *Int J Radiat Oncol Biol Phys* **40**: 787–796

Lipponen PK and Aaltomaa S (1994) Apoptosis in bladder cancer as related to standard prognostic factors and prognosis. *J Pathol* **173**: 333–339

Liver Cancer Study Group of Japan (1992) *The General Rule of the Clinical and Pathological Study of Primary Liver Cancer, 3rd edn*. Kanehara Press: Tokyo

Masuda M, Takano Y, Iki M, Asakura T, Hashiba T, Noguchi S and Hosaka M (1998) Prognostic significance of Ki-67, p53 and Bcl-2 expression in prostate cancer patients with lymph node metastases: a retrospective immunohistochemical analysis. *Pathol Int* **48**: 41–46

Molino A, Micciolo R, Turazza M, Bonetti F, Piubello Q, Bonetti A, Nortilli R, Pelosi G and Cetto GL (1997) Ki-67 immunostaining in 322 primary breast cancers: associations with clinical and pathological variable and prognosis. *Int J Cancer* **74**: 433–437

Ng IOL, Na J, Lai ECS, Fan ST and Matthew NG (1995) Ki-67 antigen expression in hepatocellular carcinoma using monoclonal antibody MIBI. A comparison with proliferating cell nuclear antigen. *Am J Clin Pathol* **104**: 313–318

 *© 1999 Cancer Research Campaign*

Nolte M, Werner M, Nasarek A, Bektas H, von Wasielewski R, Klempnauer J and Georgii A (1998) Expression of proliferation associated antigens and detection of numerical chromosome aberrations in primary human liver tumours: relevance to tumour characteristics and prognosis. *J Clin Pathol* **51**: 47–51

Sasaki K, Murakami T and Kawasaki M (1987) The cell cycle associated change of the Ki-67 reactive nuclear antigen expression. *J Cell Physiol* **133**: 579–584

Soini Y, Virkajarvi N, Lehto VP and Paakko P (1996) Hepatocellular caricinomas with a high proliferation index and a low degree of apoptosis and necrosis are associated with a shortened survival. *Br J Cancer* **73**: 1025–1030

Sugamura K, Makino M and Kaibara N (1998) Apoptosis as a prognostic factor in colorectal carcinoma. *Surg Today* **28**: 145–150

Todd D, Yang G, Brown RW, Cao J, D'Agati V, Thompson TS and Truong LD (1996) Apoptosis in renal cell carcinoma: detection by in situ end-labeling of fragmented DNA and correlation with other prognostic factors. *Hum Pathol* **27**: 1012–1017

Tormanen U, Eerola AK, Rainio P, Vahakangas K, Soini Y, Sormunen R, Bloigu R, Lehto VP and Paakko P (1995) Enhanced apoptosis predicts shortened survival in non-small-cell lung carcinoma. *Cancer Res* **55**: 5595–5602

Vesaslainen S, Lipponen P, Talja M and Syrjanen K (1994) Histological grade, perineural infiltration, tumour-infiltrating lymphocytes and apoptosis as determinants of long-term prognosis in prostatic adenocarcinoma. *Eur J Cancer* **30**: 1797–1803

Victorzon M, Roberts PJ, Haglund C, von Boguslawsky K and Nordling S (1997) Ki-67, ploidy and S phase fraction as prognostic factors in gastric cancer. *Anticancer Res* **17**: 2923–2926

Wijsman JH, Jonker RR, Keijzer R, van de Velde CJH, Cornelisse CJ and van Dierendonck JH (1993) A new method to detect apoptosis in paraffin sections: in situ end-labeling of fragmented DNA. *J Histochem Cytochem* **41**: 7–12

Wyllie AH, Kerr JFR and Curie AR (1980) Cell death: the significance of apoptosis. *Int Rev Cytol* **68**: 251–306

Yamasaki F, Tokunaga O and Sugimori H (1997) Apoptotic index in ovarian carcinoma: correlation with clinicopathologic factors and prognosis. *Gynecol Oncol* **66**: 439–448

Zhao M and Zimmermann A (1997) Apoptosis in human hepatocellular carcinoma and in liver cell dysplasia is correlated with p53 protein immunoreactivity. *J Clin Pathol* **50**: 394–400

*© 1999 Cancer Research Campaign*
